# An Evaluation of Endobronchial Ultrasound-Transbronchial Needle Aspiration (EBUS-TBNA): Molecular Diagnoses and Patient Satisfaction

**DOI:** 10.7759/cureus.101092

**Published:** 2026-01-08

**Authors:** William Wilkinson, Ben Marshall, Anindo Banerjee

**Affiliations:** 1 Southampton Faculty of Medicine, University of Southampton, Southampton, GBR; 2 Pulmonary Medicine, Southampton General Hospital NHS Foundation Trust, Southampton, GBR; 3 Respiratory Medicine, Southampton General Hospital NHS Foundation Trust, Southampton, GBR

**Keywords:** cancer immunotherapy, endobronchial ultrasound-guided transbronchial needle aspiration, mediastinal lymph node, non-small cell lung cancer, patients satisfaction

## Abstract

Background and aim: Endobronchial ultrasound-transbronchial needle aspiration (EBUS-TBNA) is a minimally invasive procedure that collects biopsies in patients with mediastinal and/or hilar lymphadenopathy, often in lung cancer or sarcoidosis. Cancerous biopsies can undergo molecular testing to identify mutations, which may be targeted by immunotherapy. The role of EBUS-TBNA in molecular outcomes has not been studied previously in the United Kingdom. This study also examines patient demographics and satisfaction to comprehensively evaluate EBUS-TBNA. This study aimed to evaluate the EBUS-TBNA service at University Hospital Southampton (UHS), focusing on patient demographics, satisfaction, diagnostic outcomes, procedural statistics, and identifying targetable mutations for immunotherapy.

Method: A total of 306 patients were studied retrospectively, and 39 were studied prospectively, using a questionnaire. Data on their characteristics, reports, diagnoses, and molecular tests were recorded and analyzed using SPSS version 29.

Results: A total of 47.12% (n = 131) patients were diagnosed with primary lung cancer, 23.38% (n = 65) with sarcoidosis, 9.71% (n = 27) with metastatic cancer, and 1.95% (n = 7) with other diagnoses, such as tuberculosis. Primary lung cancer was categorized as adenocarcinoma (52.31%, n = 68), squamous cell carcinoma (SCC) (25.38%, n = 33), and small cell carcinoma (SCLC) (16.92%, n = 23). Of the primary cancer cases, 60.31% (n = 79) underwent molecular testing. In this cohort, 29.41% (n = 20) of adenocarcinoma cases tested positive for a targetable mutation as follows: 85% (n = 17) were KRAS G12C, and 15% (n = 3) were the EGFR E19del. Overall, 15.27% (n = 20) of EBUS-TBNA procedures for primary lung cancer revealed a clinically actionable variant. Of the 39 patients surveyed, there was a significant difference between the pain experienced (median = 2/10, IQR = 4) and the expected pain (median = 5/10, IQR = 6), as assessed by a Wilcoxon signed-rank test (z = -2.91, p = 0.004). The average staff experience during the EBUS was 9.87/10 (SD = 0.47), and 55.85% (n = 21) waited <1 week for the procedure.

Conclusions: EBUS-TBNA’s role in diagnosing various conditions, especially primary lung cancer, is clear. Clinically, EBUS-TBNA provides genetic diagnoses, which can enable immunotherapy. Patient satisfaction is high, with patients expressing relief after the procedure and finding the staff exceptional.

## Introduction

Globally, 2.5 million individuals were diagnosed with lung cancer in 2022. Despite recent advancements in diagnosis/treatment, it remains the leading cause of cancer-related death [1]. The endobronchial ultrasound-transbronchial needle aspiration (EBUS-TBNA) is an integral investigation for lung cancer. EBUS-TBNA is a minimally invasive, well-tolerated method for obtaining a biopsy specimen that can undergo molecular analysis and assist in personalized patient management.

The emergence of EBUS-TBNA

Original literature published in 2002 suggests EBUS-TBNA provides greater diagnostic consistency than flexible bronchoscopy (FB). However, this was not significant [2]. Continued research and procedural advancements have improved EBUS-TBNA. In 2009, a meta-analysis found EBUS-TBNA had a "good pooled sensitivity of 0.88" [3]. Similarly, in 2013, evidence found an increased sensitivity of 0.90 [4]. This improving evidence suggests we should continue to review EBUS-TBNA to exploit all possible indications.

The National Institute for Health and Care Excellence (NICE) first published evidence on EBUS-TBNA in 2007. EBUS-TBNA is now the preferred diagnostic modality for benign and malignant mediastinal and parenchymal lesions, including lung cancer, sarcoidosis, tuberculosis, and lymphoma [5]. It has also had a significant impact on the management of these conditions [6]. EBUS-TBNA enables faster treatment, fewer complications, and improved survival [7].

Procedure of EBUS-TBNA

Before the procedure, patients’ vital signs are monitored. Intravenous sedatives, such as opioids (alfentanil), benzodiazepines (midazolam), and local anesthetics (lidocaine), are all administered. Initially, lidocaine is applied to the back of the throat when the bronchoscope is introduced.

The bronchoscope is guided anteriorly to the epiglottis, via the mouth, where the vocal cords will be visualized using the bronchoscope’s camera. Lidocaine will be administered again via the bronchoscope’s working channel, allowing access to the trachea and the mediastinal/hilar lymph nodes (2, 3, 4, 7 {subcarinal}, 10, and 11) [8].

The bronchoscope’s ultrasound head can be used to determine the lymph nodes. Its vascularity is estimated using the ultrasound color Doppler setting. This is important as excessive vascularity risks hemorrhage or specimen contamination. The needle is passed multiple times, targeting the highest-stage node on PET-CT/CT, and then further nodes are worked on backwards based on PET-CT.

However, passes will continue until the correct tissue (sometimes obtained directly from the mass) is confirmed by rapid on-site evaluation (ROSE) or until intolerance is documented. Alfentanil (maximum 1 mg) and midazolam (maximum 5 mg) are titrated to keep the patient comfortable and to avoid over-sedation. After the procedure, patients are monitored in the endoscopy ward, and most are discharged the same day.

Advantages of EBUS-TBNA

Previous staging methods, such as mediastinoscopy and thoracotomy, were notoriously invasive. EBUS-TBNA appears superior. For example, EBUS-TBNA can be repeated for the surveillance of lung cancer, whereas mediastinoscopy often leads to significant fibrosis [9]. Additionally, EBUS-TBNA reduces unnecessary thoracotomies [10], which are associated with major complications, including atelectasis and pneumonia [11].

Disadvantages of EBUS-TBNA

While EBUS-TBNA is minimally invasive, there are still risks and complications. Globally, there have been five deaths associated with EBUS-TBNA. Other complications include bleeding, bacterial pericarditis, interstitial pneumonia, sepsis, and cerebral infarction [12]. EBUS-TBNA cannot stage the whole mediastinum. It has the following three blind spots: the para-aortic, paraesophageal, and inferior pulmonary ligament nodes [3].

## Materials and methods

This study examines the role of EBUS-TBNA as a diagnostic procedure. Retrospectively, it evaluates its use in primary lung cancer, molecular testing, and the generation of clinically actionable information, alongside an analysis of patient demographics. Prospectively, the study assesses patient satisfaction through the identification of patient-reported variables, including pain and waiting time, clarity of pre- and post-procedural information, and staff experience. This study is designed as a retrospective service evaluation incorporating a prospective patient satisfaction questionnaire.

Service evaluation

The sample comprised 306 patients collected through retrospective sampling. Their inclusion was based on the criterion that they had received an EBUS-TBNA procedure, which produced a biopsy, between January 1, 2022, and January 1, 2024, at University Hospital Southampton (UHS). There were 22 exclusions. These were excluded due to the absence of a final biopsy and, therefore, any clinically actionable information. If a patient had multiple procedures within these dates, only the first EBUS-TBNA was analyzed. The list of previous EBUS-TBNA patients and their medical records was obtained via the NHS app "charts" on an encrypted trust computer in the respiratory department. The Southampton Faculty of Medicine funded this study.

In the charts, the EBUS-TBNA patient list detailed each patient’s age and sex, as well as their hospital number, which was used to access their other reports. The EBUS-TBNA report included the level of discomfort, biopsy location, the number of passes, and binary categorical variables, such as whether the positive PET-CT node was accessed. The cytological report was used to obtain final diagnoses, confirm tissue biopsies, subcategorize cancers, and identify clinically actionable information. The cytopathologist lists all molecular test results and any targetable mutations for which a drug is available within the Cancer Drug Fund. Within this study sample, only KRAS G12C and EGFR E19del were included.

Occasionally, Lung Cancer Multidisciplinary Team (MDT) notes were accessed. This was when clinical consideration was required for a final diagnosis. This was due to diagnostic uncertainty, which was often the case when biopsy material was limited or when features were nonspecific. For example, in sarcoidosis, granulomas were the only identifiable pathology.

Variable exclusions/assumptions

When recording the number of passes (i.e., the number of times a needle is inserted into the biopsy location), the location with the highest number was used. Some clinicians report a single value even if it states multiple sites were biopsied. In these cases, the location that provided the most useful diagnostic information within the cytology report was considered, and the single value was assumed to be the number of passes. For example, one location may be non-cancerous, whereas the other location is cancerous.

Cancerous diagnoses were split into primary lung cancers (cancers of the lung parenchyma/airways, such as adenocarcinoma) and metastatic cancers (cancers with a distant primary site). This classification is stated within the cytology report. Due to these exceptions, cancers such as pleural mesothelioma (cancers of the lung pleura) are excluded.

If the biopsy location was directly from the mass, the question "was the positive node on the PET-CT accessible by EBUS?" was marked as non-applicable. If the biopsy was not confirmed as the targeted tissue, the final diagnosis was also marked as non-applicable. Both these exclusions have led to "missing data" in my results section.

Data collection and analysis

Each patient was assigned an ID, and their variables were extracted and recorded using Excel. For yes/no qualitative variables, "yes" was assigned a value of 1, and "no" a value of 2. For more in-depth qualitative variables, such as the final diagnosis or the primary lung cancer subtype, additional values were assigned. After collection, the variables were transferred into the Statistical Package for the Social Sciences (SPSS version 29; Armonk, NY: IBM Corp.).

Within SPSS, percentages/frequencies were used to analyse the biopsy location, level of discomfort, binary categorical variables, final diagnoses, subcategorization, and molecular information. Distribution statistics were calculated for age and number of passes. The graph builder was also used to generate pie charts, bar charts, box plots, and distribution curves. SPSS was also used to run statistical tests, including the Wilcoxon signed-rank test.

Satisfaction questionnaire

The sample of 39 patients was collected via opportunity sampling. Their inclusion was based on the criteria that they underwent an EBUS-TBNA procedure between September 1, 2024, and December 1, 2024. The questionnaire was composed of three quantitative and four binary categorical questions (figure in appendix).

The following variables were measured: clarity of pre-/post-procedural information (ranked with a Likert scale, i.e., very clear, clear, somewhat clear, and not clear), pain experienced and expected during the EBUS-TBNA (on a scale of 0-10, 0 = no pain and 10 = worst pain), wait time experienced and expected (categorized as >1 week, one to two weeks, 2+ weeks), and experience with staff during their procedure (0-10).

Pre-procedural information refers to patients' understanding of EBUS-TBNA, whereas post-procedural information refers to symptomatic surveillance and limitations after the procedure (e.g., inability to drive for 24 h). The "time waited" variable describes the number of days between the EBUS-TBNA referral and the actual procedure date.

Data collection and analysis

Prior to EBUS-TBNA, patients were provided with information about the study, any questions were addressed, and informed consent was obtained. Post-procedurally, the patient would recover for 30-45 min, and then the questionnaire would be completed verbally. There was also a printed version for any hearing-impaired patients. Usually, the questionnaire would take no longer than 2 minutes. The questionnaire is listed in the appendix.

Each patient would be assigned a study ID, and the data would be recorded in an Excel spreadsheet (Redmond, WA: Microsoft Corp.). After collection, all the variables were transferred into SPSS. The binary categorical variables (clarity of information and wait time experienced/expected) were analyzed using percentages and bar graphs. Quantitative variables (pain experienced/expected and staff experience) were analyzed using frequencies/distribution curves.

Statistical tests

To determine patient satisfaction, the difference between their experienced/expected variables was explored to see if EBUS-TBNA exceeded their expectations. Two Wilcoxon signed-rank tests were conducted at p = 0.05 and a confidence interval of 95% to determine whether there was a significant difference between the pain and wait experienced/expected.

Protocol validation

Ethics and Research Governance Office (ERGO) approval was applied for in May and granted in June 2024. This included the completion and submission of seven documents detailing the study methods, sample, and preliminary questionnaire drafts. Dr. Marshall reviewed these documents prior to ERGO submission.

## Results

Service evaluation

Introduction

**Figure 1:**
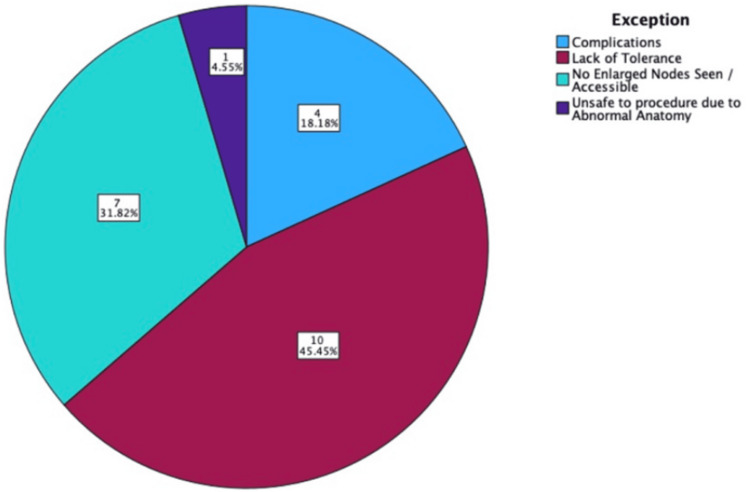
Pie chart of EBUS exclusions. EBUS: endobronchial ultrasound

Between January 1, 2022, and January 1, 2024, 306 EBUS-TBNA procedures were performed at UHS. Twenty-two EBUS-TBNA cases were excluded (Figure 1). These were due to a lack of tolerance, the absence of abnormal nodes on ultrasound, complications, or unusual anatomy, accounting for 10 (45.45%), 7 (31.82%), 4 (18.18%), and 1 (4.55%) exclusions, respectively.

A lack of tolerance would often be reported as excessive coughing/restlessness despite maximal sedation. During the EBUS-TBNA, if the suspected pathological node appeared normal on ultrasound, the EBUS-TBNA would be abandoned. Complications included hypoxia (with one patient dropping as low as 70% oxygen saturation), as well as one patient with a perceived increased risk of mediastinitis due to lymph nodes with "cystic features." Abnormal anatomy was reported in one case, where the paratracheal node was too high to attempt a biopsy safely. One EBUS-TBNA was converted to bronchoscopy due to upper lobe rigidity, and the smaller needle used in bronchoscopy was required.

Patient Demographics

**Figure 2:**
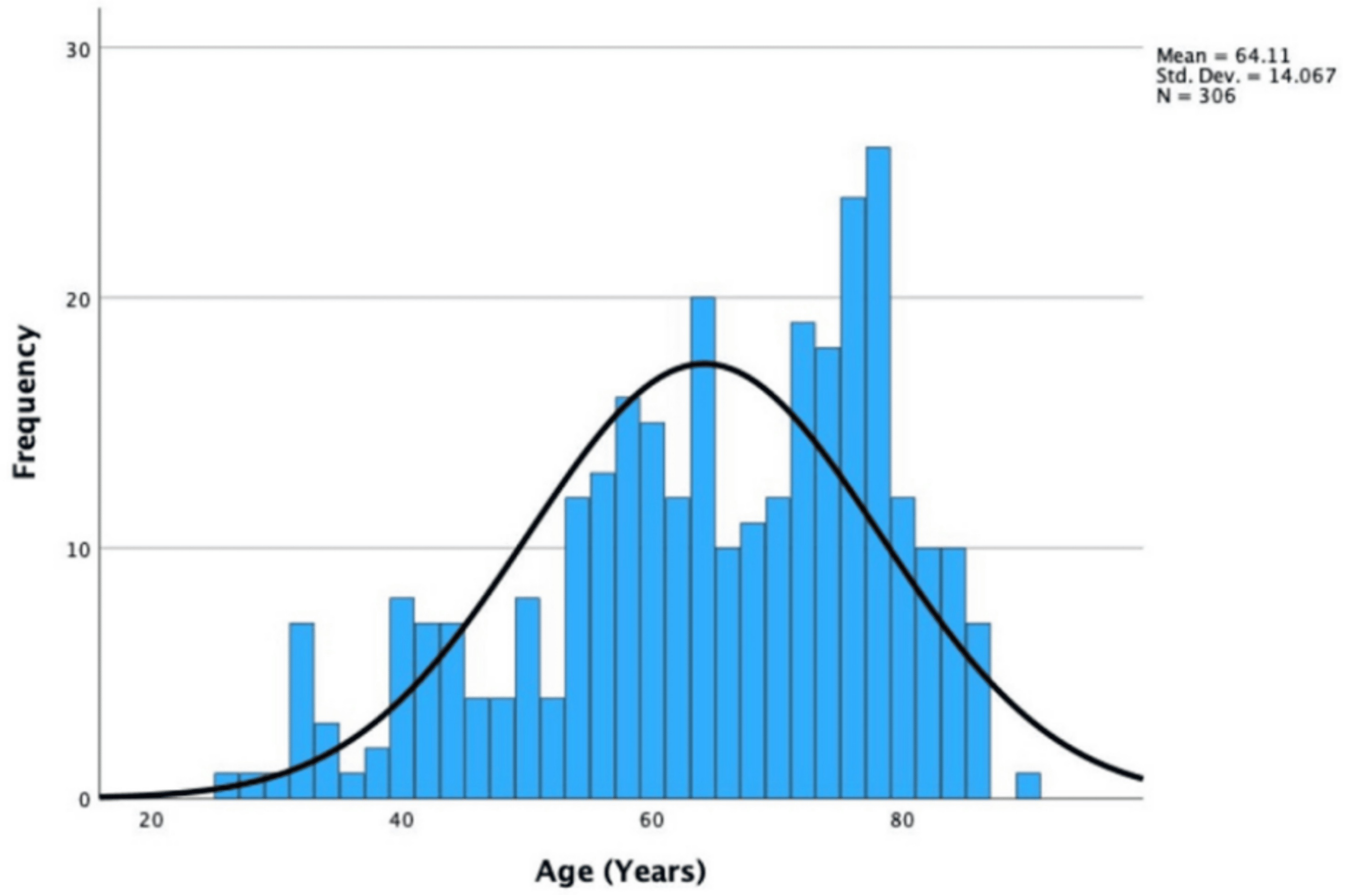
Histogram showing the age of EBUS-TBNA patients. EBUS-TBNA: endobronchial ultrasound-transbronchial needle aspiration

Of the 306 EBUS patients, 53.59% were male (n = 164), and 46.41% were female (n = 142). The median age was 66 years (IQR = ±21.00). Age was non-parametric, with skewness of -0.63 and a standard error of 0.14, which was significant (-0.63/0.14 = -4.50 >±1.96). These statistics are displayed in Figure 2.

EBUS Statistics

**Figure 3:**
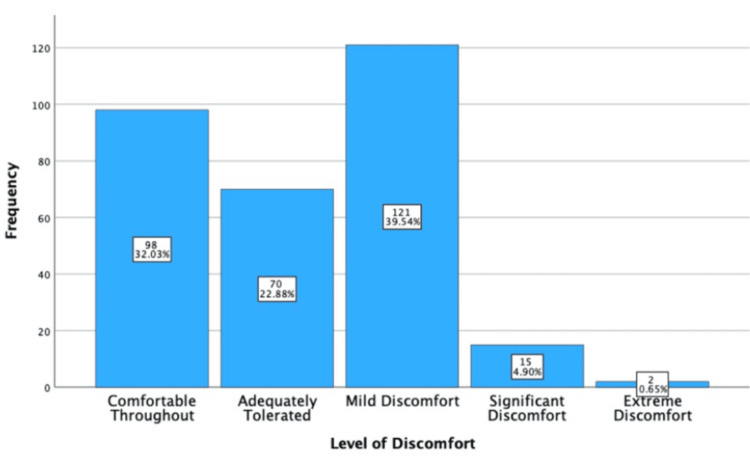
Simple bar chart outlining the clinician-reported level of discomfort during EBUS-TBNA. EBUS-TBNA: endobronchial ultrasound-transbronchial needle aspiration

The level of discomfort is recorded by the clinician using a five-point Likert scale (i.e., comfortable {1}, adequately tolerated {2}, mild {3}, significant {4}, and extreme discomfort {5}). 32.03% (n = 98) experienced comfort throughout, 22.88% (n = 70) of patients tolerated the EBUS adequately, and 39.54% (n = 121) of patients experienced mild discomfort. Only 5.55% (n = 17) experienced either significant or extreme discomfort. These results are displayed in Figure 3*.*

**Figure 4:**
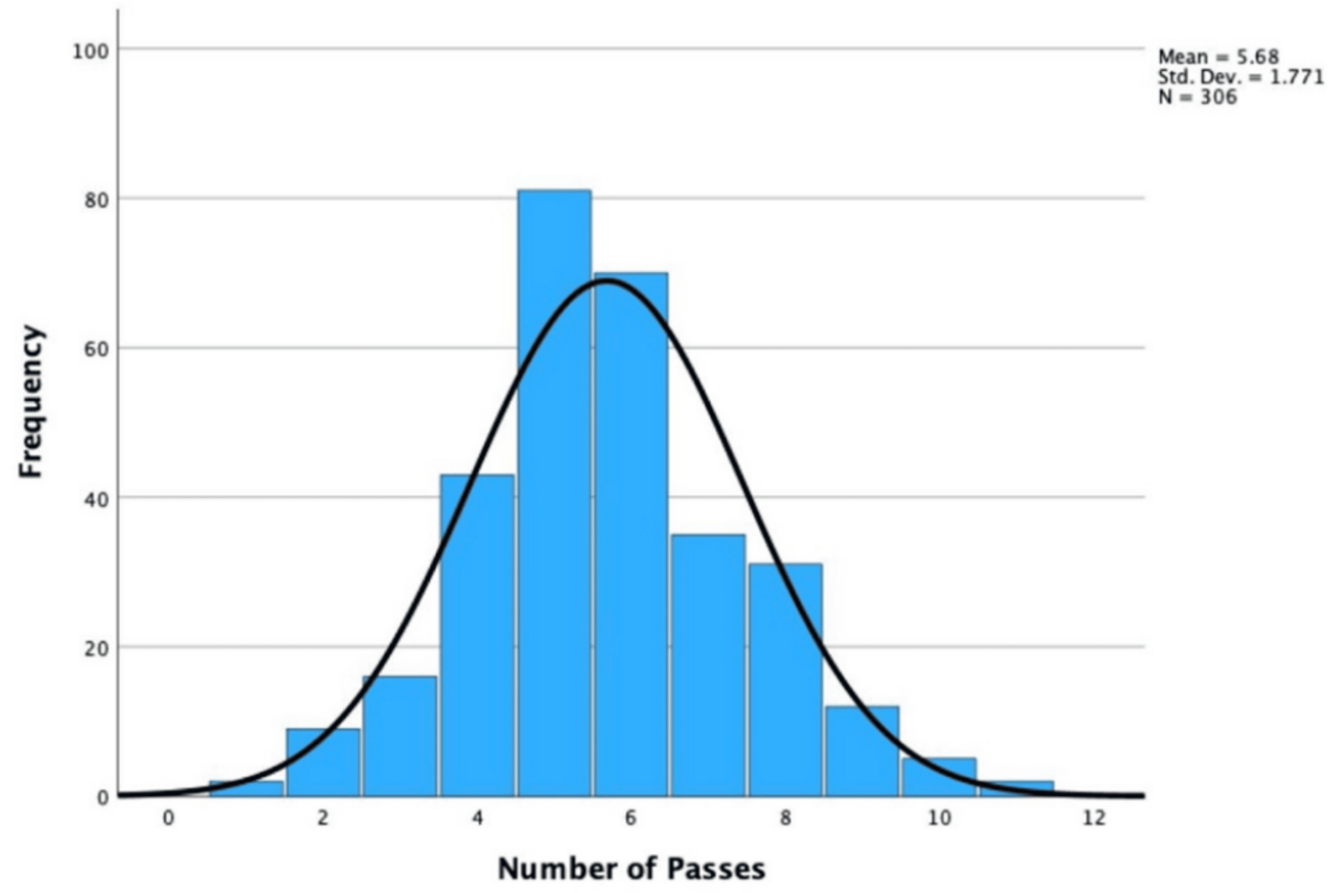
Histogram of the Number of Passes during the EBUS-TBNA EBUS-TBNA: endobronchial ultrasound-transbronchial needle aspiration

The mean number of passes was 5.68 (SD = ±1.77) and was parametric. This is because the skewness value (0.25) and the standard error (0.14) were not statistically significant (0.25/0.14 = 1.81 < ±1.96). The distribution of these statistics is explored in Figure 4.

In 95.10% (n = 273) of EBUS cases, the EBUS-TBNA could access the positive node identified on the PET-CT/CT performed before the procedure. A total of 95.80% (n = 293) of biopsies matched the tissue type at the reported biopsy site, and in 91.20% (n = 279) of cases, this biopsy provided sufficient material for a final diagnosis. These statistics are shown in Table 1.

**Table 1:** Variables demonstrating diagnostic ability of EBUS-TBNA. EBUS-TBNA: endobronchial ultrasound-transbronchial needle aspiration

Variables	Yes	No	Percentage
Could the EBUS-TBNA access the positive node on the PET-CT?	273	14	95.10
Did the biopsy match the tissue type of the location sampled?	293	13	95.80
Did the biopsy provide enough material for a final diagnosis?	279	27	91.20
Did they receive a final diagnosis?	279	27	91.20

**Figure 5:**
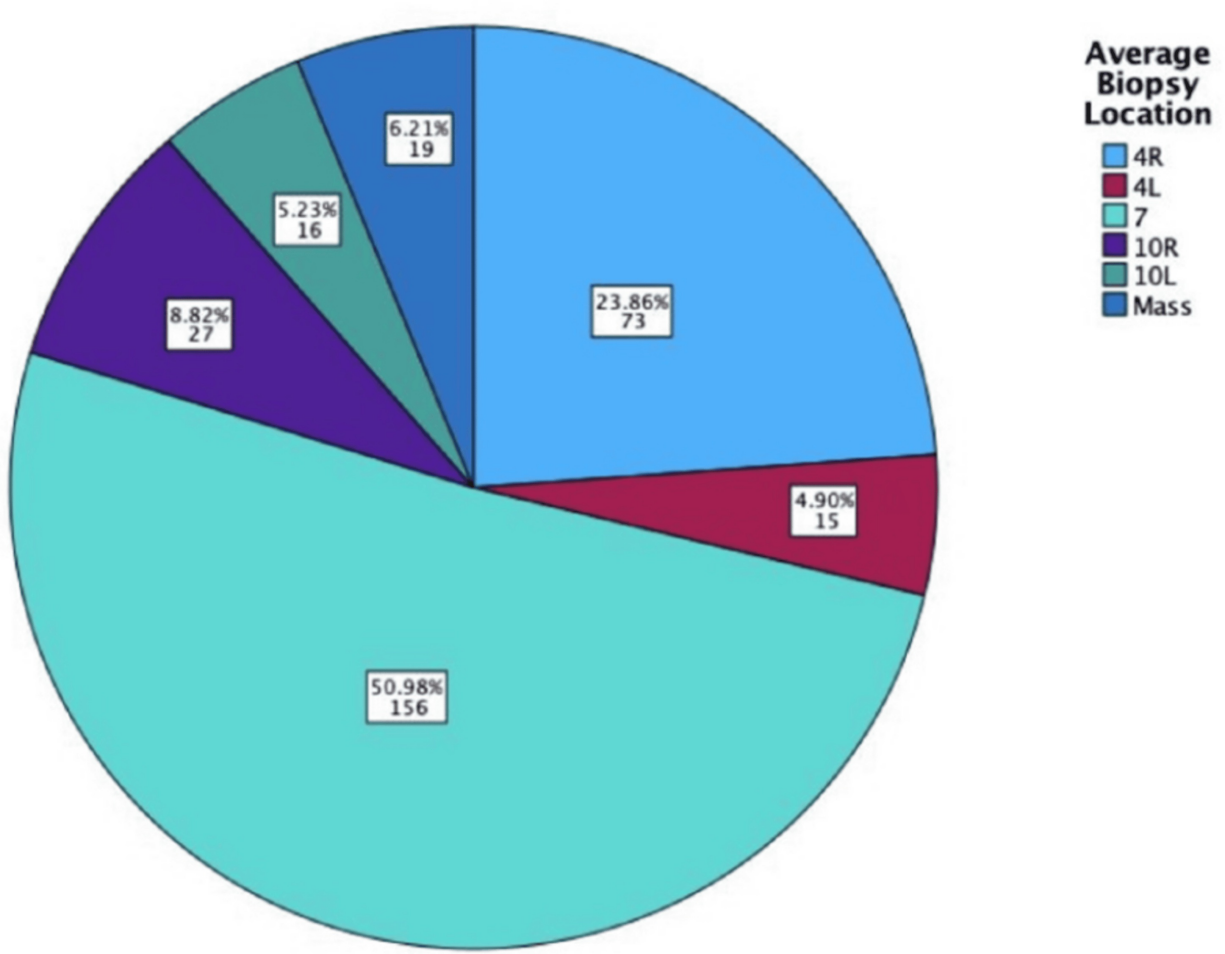
Pie chart showing the EBUS-TBNA biopsy locations. EBUS-TBNA: endobronchial ultrasound-transbronchial needle aspiration; 4R: right paratracheal node; 4L: left paratracheal node; 7: subcarinal; 10R: right hilar nodes; 10L: left hilar nodes

The locations of biopsy were the right paratracheal node (4R; 23.86%, n = 73), the left paratracheal node (4L; 4.90%, n = 15), the subcarinal node (7; 50.98%, n = 156), the right hilar nodes (10R; 8.82%, n = 27), the left hilar nodes (10L; 5.23%, n = 16), and biopsies taken directly from a mass (6.21%, n = 19). These are visually displayed in Figure 5.

Factors Affecting the Likelihood of a Final Diagnosis

Females were slightly more likely to receive a final diagnosis (92.96%, n = 129) compared to males (90.85%, n = 149). Patients who received a final diagnosis also had a greater number of passes during their EBUS-TBNA (mean = 5.69 {SD = ±1.75} in those who received a final diagnosis and 5.56 {SD = ±1.99} in those who did not).

Older individuals were slightly more likely to receive a final diagnosis (median = 67.00; IQR = ±21.00) than those who did receive a diagnosis (median = 61.00; IQR = ±16.00) or those who did not (median = 61.00; IQR = ±16.00). Age had an impact on the diagnostic outcome category. The median age of a primary lung cancer patient was 68.00 (IQR = ±17.00) years old, and for sarcoidosis, it was 61.00 (IQR±16.00). The remaining data are presented in Tables 2, 3. The location of the biopsy did have an impact on the likelihood of receiving a final diagnosis. The left hilar nodes and mass biopsies yield a 100% likelihood of a final diagnosis (n = 16 and 19, respectively). The largest sample, the subcarinal node (n = 156), has a 90.38% chance of a final diagnosis. The remaining data are also shown in Table 2.

**Table 2:** Factors affecting the likelihood of a final diagnosis.

Variables	Diagnosis confirmed	Diagnosis not confirmed
Number of passes (mean±SD)	5.69±1.75	5.56±1.99
Age in years (median±IQR)	67.00±21.00	61.00±16.00
Effect of sex		
Female (%, n)	90.85 (129)	9.15 (13)
Male (%, n)	92.96 (149)	7.04 (15)
Effect of biopsy location		
Right paratracheal node (4R) (%, n)	88.89 (64)	11.11 (8)
Left paratracheal node (4L) (%, n)	80.00 (12)	20.00 (3)
Subcarinal node (7) (%, n)	90.38 (141)	10.64 (15)
Right hilar nodes (10R) (%, n)	96.30 (26)	3.70 (1)
Left hilar nodes (10L) (%, n)	100 (16)	0 (0)
Mass biopsies (%, n)	100 (19)	0 (0)

**Table 3:** Number and ages of different EBUS-TBNA diagnoses. EBUS-TBNA: endobronchial ultrasound-transbronchial needle aspiration

Condition diagnosed	Percentage (n)	Age in years (median±IQR)
Primary lung cancer	47.12 (131)	68.00±17.00
Sarcoidosis	23.38 (65)	57.00±23.50
Tuberculosis	1.80 (5)	35.00±18.50
Metastatic cancer	9.71 (27)	73.00±16.00
Reactive lymph node	0.72 (2)	73.50±0.00
No atypical cells	17.27 (48)	71.50±17.25
Total	100 (306)	66.00±21.00

Final Diagnostic Information

**Figure 6:**
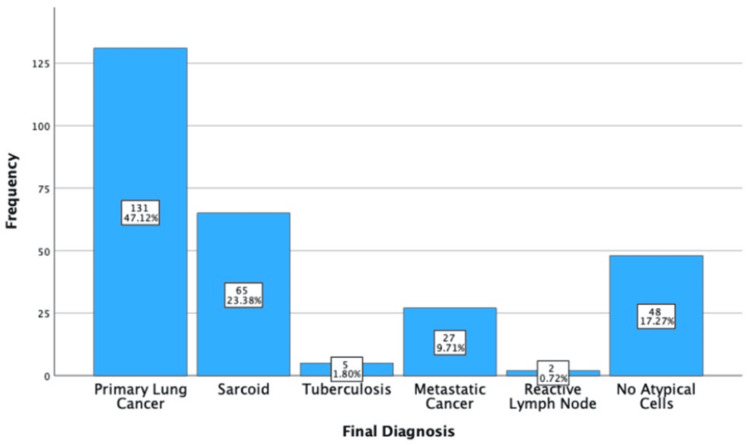
Simple bar chart showing the final diagnostic results.

A total of 278 individuals received a final diagnosis. These were primary lung cancer (47.12%, n = 131), sarcoid (23.38%, n = 65), metastatic cancer (9.71%, n = 27), tuberculosis (1.80%, n = 5), and reactive lymph nodes (0.72%, n = 2). Among the cohort, 17.27% (n = 48) had a final diagnosis of "no atypical cells," which indicates a biopsy with no pathology. These statistics are shown in Table 3 and Figure 6.

Primary Lung Cancer Subtyping Statistics

**Figure 7:**
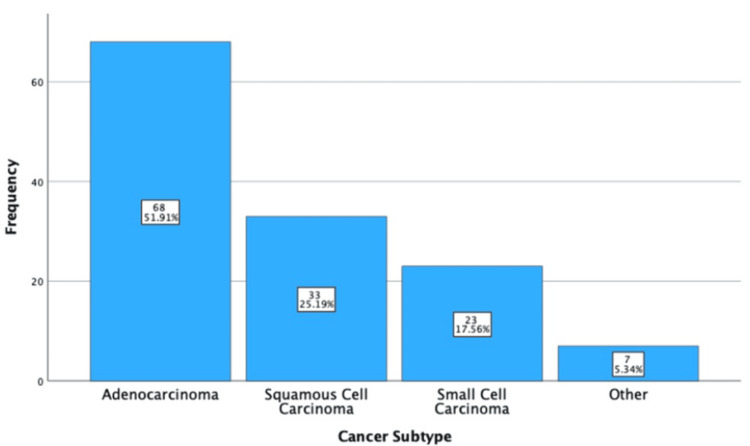
Simple bar chart showing the subcategorization of primary lung cancer.

Of the 131 primary lung cancers, 51.91% (n = 68) were adenocarcinoma, 25.19% (n = 33) were squamous cell carcinoma, 17.56% (n = 23) were small cell carcinoma, and seven of them did not fit into these criteria (termed "other"). Of the other carcinomas, there were three undifferentiated, two neuroendocrine, one pleomorphic, and one carcinoid carcinoma. These results are displayed in Figure 7.

Molecular Testing

A total of 60.31% (n = 79) of all primary lung cancers underwent molecular testing, and 25.32% (n = 20) of those tested provided clinically actionable information. Among the cohort, 95.59% (n = 65) of adenocarcinomas underwent molecular testing, with only three not undergoing testing. This was due to the sample containing too little material, with the cytology report stating the impossibility of next-generation sequencing (NGS). Only 24.24% (n = 8) of squamous cell carcinomas underwent molecular testing, and no small cell carcinomas underwent testing. Of the other carcinomas, 85.71% (n = 6) underwent testing.

Obtaining Clinically Actionable Information

Only adenocarcinoma provided clinically actionable information, with 29.41% (n = 20) testing positive. Of these, 85% (n = 17) were the KRAS G12C variant and 15% (n = 3) were the EGFR E19del variant. However, these were not the only variants tested. The KRAS Gly12Ala variant tested positive in one case, and Mesenchymal-Epithelial Transition (MET) amplification in two cases. However, these were omitted from Table 4, as no NICE-approved treatments exist for these variants. However, it must be noted that despite exclusion, these variants may unlock the accessibility of new treatments in the future. All this data are shown in Table 4.

**Table 4:** Molecular testing of different cancer subtypes and their outcomes.

Cancer subtype	Percentage of primary lung cancer diagnoses	Underwent molecular testing, % (n)	Provided a clinically actionable mutation	KRAS G12C positive	EGFR E19del positive
Adenocarcinoma, % (n)	51.910 (68)	95.59 (65)	29.41 (20)	85 (17)	15 (3)
Squamous cell carcinoma, % (n)	25.19 (33)	24.24 (8)	0 (0)	0 (0)	0 (0)
Small cell carcinoma, % (n)	17.56 (23)	0 (0)	0 (0)	0 (0)	0 (0)
Other	5.34 (7)	85.71 (6)	0 (0)	0 (0)	0 (0)
Total	131	60.31 (79)	15.27 (20)	12.98 (17)	2.29 (3)

Factors Affecting the Likelihood of a Clinically Actionable Diagnosis

**Figure 8:**
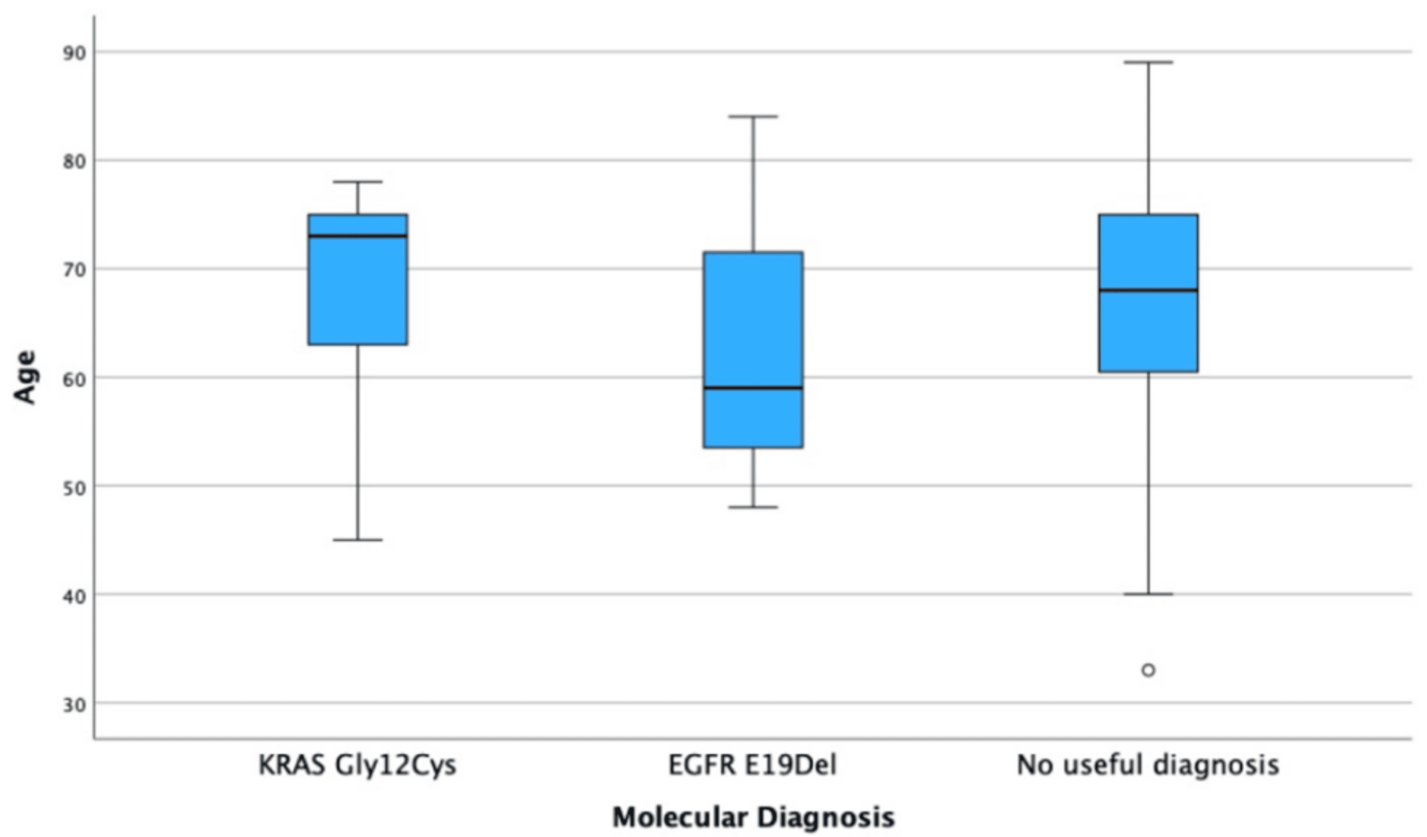
Box plot of the effect of age on the likelihood of a clinically actionable diagnosis.

Those who tested positive with a G12C variant tended to be older (median = 73.00 {IQR = ±13.00}) than those with an E19del variant (median = 59.00 {IQR = ±27.00}). These are displayed in Table 5 and Figure 8.

**Table 5:** Factors affecting the likelihood of a useful diagnosis after molecular testing has been performed.

Variables	Tested positive for a KRAS G12C variant	Tested positive for an EGFR E19del variant	Underwent testing with no clinically actionable information obtained
Age in years (median±IQR)	73.00±13.00	59.00±27.00	68.00±14.75
Female, % (n)	23.01 (12)	3.85 (2)	73.08 (38)
Male, % (n)	16.67 (5)	3.33 (1)	80 (24)

Prospective Satisfaction Questionnaire Results

Thirty-nine participants answered the questionnaire. There were three omissions due to a language barrier and the inability to obtain a translator.

Clarity of Information

A total of 87.18% (n = 34) reported pre-procedural information as "very clear," 10.26% (n = 4) as "clear," and 2.56% (n = 1) as "somewhat clear," which was due to frustration over a delay in the production of the EBUS information booklet. A total of 84.62% (n = 33) reported post-procedural information as "very clear," 5.13% (n = 2) as "clear," and 10.26% (n = 4) as "somewhat clear." This information is shown in Table 6.

**Table 6:** Reported clarity of EBUS-TBNA information. EBUS-TBNA: endobronchial ultrasound-transbronchial needle aspiration

Variables	Pre-procedural information	Post-procedural information	Difference
Very clear, % (n)	87.18 (34)	84.62 (33)	-2.56 (-1)
Clear, % (n)	10.26 (4)	5.13 (2)	-5.13 (-2)
Somewhat clear, % (n)	2.56 (1)	10.26 (4)	+7.70 (+3)
Not clear, % (n)	0 (0)	0 (0)	+0 (+0)

Pain Experience

**Figure 9:**
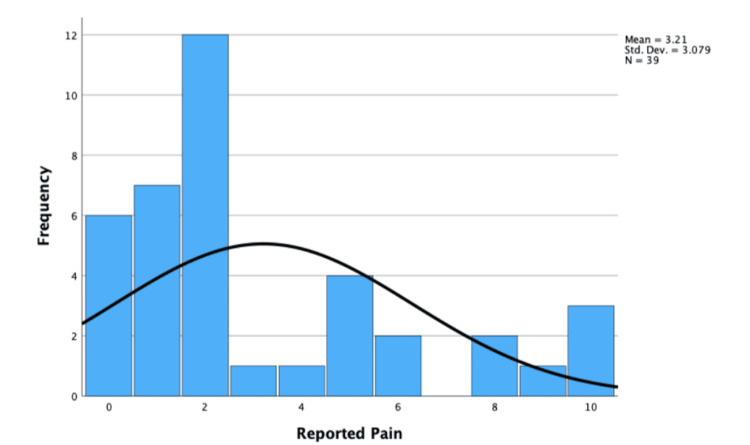
Histogram displaying reported experienced pain during EBUS-TBNA. EBUS-TBNA: endobronchial ultrasound-transbronchial needle aspiration

**Figure 10:**
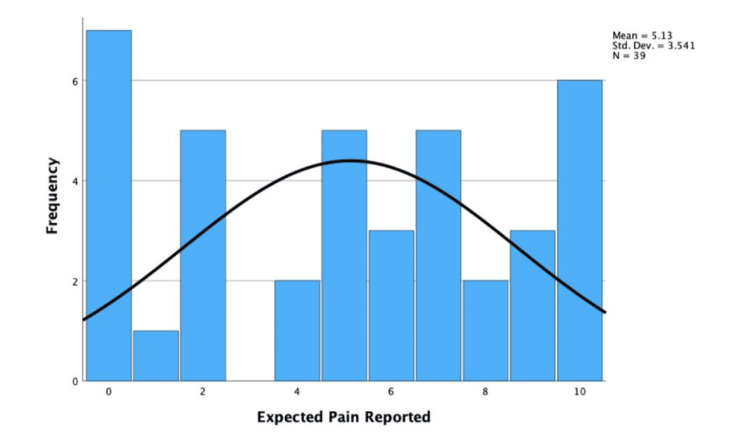
Histogram displaying reported expected pain during EBUS-TBNA. EBUS-TBNA: endobronchial ultrasound-transbronchial needle aspiration

The median reported pain experienced was 2.00 (IQR ± 4.00), and the distribution was non-parametric. This was because the skew of 1.07 and standard error of 0.38 were significant (1.07/0.38 = 2.82 > ±1.96). The mean expected pain was reported as 5.13 (SD = ±3.54), which was parametric. This was because the skewness was -0.14 and the standard error was 0.38, which were not statistically significant (-0.14/0.38 = -0.37 < ±1.96). These are shown in Figures 9, 10.

The Wilcoxon signed-rank test found a statistically significant difference between experienced and expected pain (0.05 > 0.004), and the null hypothesis was rejected. Therefore, the difference between experienced and expected pain can be concluded as statistically significant.

Waiting Times

A total of 53.85% (n = 21) reported <1 week, 33.3% (n = 13) as one to two weeks, and 12.82% (n = 5) as >2 weeks in waiting time. This was non-parametric as the skew of 0.81 and the standard error of 0.38 were significant (0.81/0.38 = 2.13 > ±1.96). When asked about expected waiting time, 41.03% (n = 16) reported <1 week, 35.90% (n = 14) one to two weeks, and 23.08% (n = 9) >2 weeks. This was parametric, as the skew of 0.34 and the standard error of 0.38 were insignificant (0.34/0.38 = 0.90 < ±1.96). These results are shown in Table 7.

**Table 7:** Experienced and expected waiting times for EBUS-TBNA. EBUS-TBNA: endobronchial ultrasound-transbronchial needle aspiration

Variables	Experienced	Expected	Difference
<1 week, % (n)	53.85 (21)	41.03 (16)	-12.82 (5)
1-2 weeks, % (n)	33.3 (13)	35.90 (14)	+2.6 (1)
>2 weeks, % (n)	12.82 (5)	23.08 (9)	+10.26 (4)

The Wilcoxon signed-rank test found the difference was insignificant (0.078 > 0.05), and the null hypothesis was accepted. Therefore, the difference between expected and experienced wait times can be concluded as insignificant.

Staff Experience

The median response was 10 (IQR = ±0). However, the consistently high rating likely reflects the pivotal role of staff professionalism, communication, and reassurance during the EBUS-TBNA, an invasive and potentially anxiety-provoking procedure for patients. Supportive staff interactions may significantly influence patient comfort, perceived safety, and overall tolerance of the procedure, independent of technical outcomes. As such, staff behavior represents a key component of the patient experience in EBUS services and may positively shape patients' overall perception of care.

## Discussion

Service evaluation

Exceptions

Only 3.05% (n = 10) of EBUS-TBNA were excluded due to lack of tolerance, and 1.22% (n = 4) due to complications, suggesting that the procedure is well tolerated and minimally invasive.

Patient Demographics

The large IQR in age highlights the frequent use of EBUS-TBNA. The distribution of ages across different EBUS-TBNA indications varied significantly. The most common indication, primary lung cancer, had a higher median age than the second most common, sarcoidosis. However, the small sample size for the rarer indications may limit the ability to report medians and IQRs.

EBUS statistics

Level of Discomfort

Given that 94.44% of individuals did not experience significant/extreme discomfort and that the complication rate was low, EBUS-TBNA can be considered adequately tolerated. However, the "level of discomfort" is clinician-reported based on the patient’s appearance during the procedure, introducing subjectivity. We cannot conclude the level of inter-observer bias or construct validity. Standardization training via a Virtual Learning Environment (VLE) may be required to ensure inter-rater reliability and to ensure that the tolerability information provided to patients is accurate.

Number of Passes

While the average number of passes increased with a final diagnosis, the increase was minimal (+0.13). Confounding variables, including differences between clinicians and patients, are significant and affect the study's internal validity. Some clinicians require more passes than others, and differences in reporting styles necessitate assumptions. Additionally, the control of the rapid on-site evaluation (ROSE) cytopathologist over the number of passes may increase the number of passes unnecessarily. Patient heterogeneity, including anatomical and tolerability differences, complicates the data. Unlike the level of discomfort, this variable is more clinically significant and only slightly affects the patient outcomes. If the correct biopsy is confirmed and molecular testing is completed, this variable is irrelevant.

Binary Categorical Variables

The majority of EBUS-TBNA accessed the positive node on PET-CT-CT, matched the biopsy location, and provided a final diagnosis, which indicates the appropriate/effective use of EBUS-TBNA. For the cases in which the positive node was not accessed, there may have only been a single, small, and distal positive node, which may also be inaccessible to other non-surgical techniques.

Whether the biopsy matched the reported location was a measure of the clinician’s skill/communication with the ROSE pathologist to ensure the correct tissue had been sampled. However, histopathologists occasionally make mistakes that lead to the misdiagnosis of a biopsy [13]. Although this is occasional, the sample size may have been large enough for this to occur. Continued auditing of cytopathology may be required to reduce the need for unnecessary secondary procedures.

Three adenocarcinoma biopsies provided enough material for a diagnosis but not for molecular classification. Although this accounts for only 4.41% of adenocarcinomas, it may become more clinically relevant as molecular therapies improve.

Location of Biopsies

EBUS-TBNA accesses a variety of nodes successfully, with five different nodes sampled. Also, it appears optimized for patient safety. The subcarinal node was the most common node sampled, which could be due to its large size and accessibility, reducing the risk of complications. 4R was sampled 58 more times than 4L. While they are the same size, the proximity of 4L near large venous structures, including the left brachiocephalic vein, increases the likelihood of complications [14].

Similarly, right-sided thoracic nodes are more accessible due to the shallower angle and wider lumen of the right primary bronchus. By prioritizing these nodes in a clinical setting, you reduce the likelihood of complications such as pneumothorax. While mass biopsies provided a 100% chance of final diagnosis, this relied on the position of the mass being in close enough proximity to the bronchus without corroding the inner mucosa. If bronchoscopy had been used, samples from the 4 L, 10 L, and mass biopsy groups would each have included fewer than 19 cases, meaning the results may not be generalizable. A larger sample size would be required when developing guidelines on node prioritization to optimize diagnostic success and reduce complications.

Likelihood of a Final Diagnosis

Males may be more likely to receive a final diagnosis (+2.11%); however, this may be due to a lack of proportional representation within the sample. Older individuals were more likely to receive a diagnosis (+6.00 years), due to the distribution of ages within the different diagnoses made.

Older individuals were more likely to receive a diagnosis of cancer compared to sarcoidosis/TB. Sarcoidosis/TB have non-specific pathological features, such as granulomas, and pathologists often struggle to diagnose this with material alone. This study's diagnostic data may lack external validity and generalizability, as the sample is Wessex-based, and this population may have specific pathological/diagnostic trends that are not representative of other populations.

Diagnostic Information

EBUS-TBNA plays a clear role in the diagnosis of respiratory/cancerous pathology. There were five pathological diagnoses, and 17.27% (n = 48) of individuals showed normal nodes, with no abnormalities seen. As expected, cancer was the most frequent diagnosis, followed by sarcoidosis. However, the sample was not large enough to show all the possible indications [15].

Cancer Subtyping

Unusually, no large cell carcinomas were diagnosed, as they represent 10% of non-small cell lung cancer (NSCLC). However, there was an increase in adenocarcinoma (+17.11%), squamous cell carcinoma (SCC) (+3.44%), and small cell carcinoma (SCLC0 {+5.56%}). This shift is likely due to differences in subtype frequency between populations; for example, Asian men are more likely to be diagnosed with adenocarcinoma compared with men in Europe [16].

Undifferentiated carcinoma was classified as "other" as only the first EBUS-TBNA was considered. These patients may undergo another diagnostic procedure, which could classify the carcinoma within the subtypes. This assumption reduces the internal validity.

Molecular Testing

No small cell carcinoma underwent molecular testing because targetable mutations are uncommon [17]. Only 24.24% of SCC underwent molecular testing, as G12C and E19del are rare. However, the cytopathologist at UHS suggested that molecular testing for SCC is becoming more routine.

The present study demonstrated an increased frequency of G12C (+9-12%) and a large decrease in E19del (-19.07%) within the adenocarcinoma sample. The external validity of these results is questionable, as the small sample of E19del is too small to be generalizable. Also, Asians have a much larger rate of EGFR mutations compared to Caucasians, and the sample may represent a causation-focused sample [16]. To improve generalisability, future studies would need to account for nationality within a larger, more representative sample. Nonetheless, these findings reinforce the continued importance of EBUS-TBNA in generating clinically actionable diagnostic outcomes.

Satisfaction questionnaire

Clarity

Pre-procedural information is reported as very clear, and the patient’s consent can be seen as appropriate and well-informed. However, these statistics were patient-reported and may have been influenced by their emotions/environment during the questionnaire. For example, the only report of "somewhat clear" was due to the frustration over a delay in the production of the information booklet. Additionally, the presence of other patients may elicit social desirability bias, as patients may not want to appear unintelligent by admitting they cannot recall recently delivered information. The patient’s level of education and understanding should be controlled for in a repeat study.

There was an insignificant decrease in the reported clarity of post-procedural information. This may be because the information is provided after EBUS-TBNA, when the anterograde amnestic effects of midazolam may reduce the accuracy of their recollection [18].

Pain

Patients would often struggle to suggest a number between 0 and 10 and suggest EBUS-TBNA is "uncomfortable." The low pain experienced and significant positive skew suggest that EBUS-TBNA is not painful, supporting the appropriate use of sedatives. The significant difference between experienced/expected pain also suggests patients are often anxious and feel relieved after the EBUS-TBNA.

Expected pain was moderate (mean = 5.13), supporting the accuracy of the pre-procedural information on pain. Anxiety is subjective; therefore, inter-patient variability must be considered. Some patients stated they’d had an EBUS-TBNA before and said they experienced pain matching the expected. Different clinicians use different doses of sedatives, which likely affects the reported pain. The Likert scale is also unreliable, as patients have different ideas of what a "10/10 pain" is. To improve validity, the visual analog scale (VAS) and a larger sample could be used.

Waiting Times

Patients struggled to provide specific dates due to the sedatives, which reduced the construct validity. A total of 87.15% of individuals fit within the two-week cancer pathway. The other individuals may have had non-cancerous conditions, such as sarcoidosis, which has a lower mortality rate [19]. While insignificant, there is an increase in the wait time expected. Perhaps due to the patient’s prior options/knowledge of NHS wait times, or because of gratitude/lingering euphoric sedative effects causing the exaggeration of expected times.

The non-significant difference may be because the clinician who originally detailed the EBUS-TBNA may have told them the expected wait. Some patients also expected a quicker appointment, and this may have been due to anxiety/frustration about suspected cancer.

Staff Experience

The results suggest the staff were exceptional during the EBUS-TBNA. This variable, in particular, is not valid due to social desirability bias. The presence of the researcher and surrounding clinicians or patients may have exerted pressure on respondents to provide more favorable ratings.

Changes in a Repeat Study

The small sample size and the Wessex-based population limit generalizability. Confounding variables and inter-patient variability are too significant to ignore, and caution may be needed when drawing mass conclusions based on this data. There is a lack of objective measures, with both clinician and patient reports, and this limits the validity of the study.

If this study were repeated, a larger national sample should be used. Additionally, training in EBUS-TBNA reporting could be provided to clinicians in advance, thereby reducing the number of assumptions required. The questionnaire should be completed in a private setting, or perhaps over the phone, a few days after the procedure, to minimize social desirability bias and the antegrade amnesic effects of sedation.

## Conclusions

EBUS-TBNA is a well-tolerated and minimally invasive diagnostic procedure that can access many locations safely and investigate various conditions. EBUS-TBNA also plays a vital role in the molecular analysis and subsequent patient management of non-small cell lung cancer, which may help to improve patient outcomes. As more immunotherapy drugs are included within the cancer drug fund, the outlook of EBUS-TBNA will continue to improve. Also, patients are satisfied with the service, potentially improving their cancer care.

## Figures and Tables

**Figure 11:**
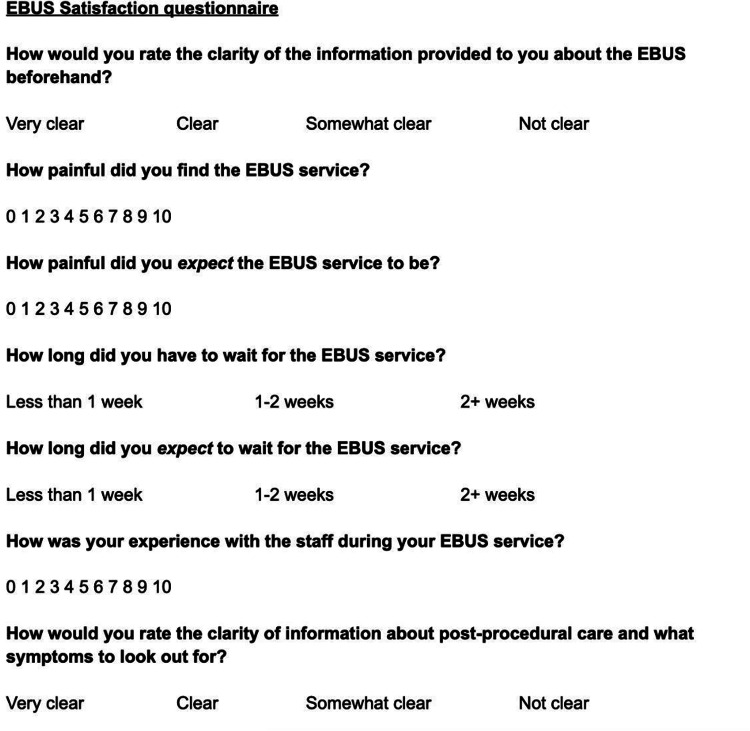
EBUS satisfaction questionnaire. EBUS: endobronchial ultrasound service
